# Associations Between Gut Microbiome Enterotypes and Body Weight Change During Whole Milk Consumption

**DOI:** 10.3390/nu18040563

**Published:** 2026-02-09

**Authors:** Panpan Qin, Lelde Berzina, Nina Rica Wium Geiker, Karoline Sandby, Thure Krarup, Karsten Kristiansen, Faidon Magkos

**Affiliations:** 1 Laboratory of Integrative Biomedicine, Department of Biology, University of Copenhagen, 2100 Copenhagen, Denmark; 2 Department of Nutrition, Exercise and Sports, University of Copenhagen, 1958 Frederiksberg, Denmark; 3 Centre for Childhood Health, 2300 Copenhagen, Denmark; 4 Department of Endocrinology, Copenhagen University Hospital Bispebjerg and Frederiksberg, 2400 Copenhagen, Denmark

**Keywords:** personalized nutrition, whole milk, obesity, enterotype, taurine, *Streptococcus thermophilus*

## Abstract

**Background:** Evidence is accumulating that gut bacterial communities modulate the outcome of dietary interventions. **Objective:** To assess how gut microbial enterotypes correlate with obesity-related outcomes during one month of whole milk consumption. **Methods:** This post hoc analysis used data from a previously published trial, which included a lead-in phase during which men with abdominal adiposity replaced habitual dairy product consumption with 400 g/day of whole milk for one month. We compared body weight, urinary metabolites, fecal metabolites, and gut microbiome composition and function based on shotgun metagenomic sequencing at the beginning and at the end of the lead-in phase between individuals with the two most prevalent enterotypes, the *Bacteroides*1 (B1) enterotype (*n* = 24) and the *Ruminococcaceae* (R) enterotype (*n* = 38). **Results:** Individuals with the B1 enterotype, but not those with the R enterotype, exhibited decreases in body weight and the relative abundance of *Streptococcus thermophilus*. Multiple linear regression analysis identified enterotype as a strong predictor of body weight change (*p* = 0.0034). In addition, urinary taurine level change was positively associated with body weight change in B1 individuals, not in R individuals. **Conclusions:** Our findings reveal an enterotype-specific response to an identical dietary modification, underscoring the value of integrating enterotype information into nutrition-intervention design and personalized nutrition strategies.

## 1. Introduction

Overweight and obesity remain pressing global health concerns, contributing substantially to the burden of metabolic disorders, such as insulin resistance, hypertension, and hepatic steatosis [1,2]. Among various treatment strategies, dietary interventions have been widely exploited. Although approaches like low-carbohydrate diets [3,4], the Mediterranean diet [3,5,6], and intermittent fasting [7,8] have demonstrated efficacy, their long-term effectiveness remains limited in real-world settings and varies widely between individuals, partly because of differing adherence.

In our earlier randomized controlled trial (RCT) in men with abdominal adiposity, participants underwent a six-month intervention with different dairy products, preceded by a one-month standardizing lead-in phase [9,10]. During the lead-in phase, participants replaced habitually consumed dairy products with whole milk (400 g/day). By design, no significant group-level changes in body weight were anticipated to occur during the lead-in phase, but we observed significant inter-individual variability. This, together with emerging evidence implicating the gut microbiome in mediating host–diet interactions [11,12,13], led us to hypothesize that the gut microbial community structure, the so-called enterotype, may be associated with body weight changes during the lead-in phase.

Gut microbial enterotypes were originally proposed as three major community types driven by: *Prevotella* (P enterotype), *Bacteroides* (B enterotype), and *Ruminococcus* (R enterotype) [14]. Subsequent larger studies refined this framework, including subdivision of the B enterotype into B1 and B2, with B2 associated with lower microbial diversity and higher levels of inflammatory markers [15]. The original notion of discrete community clusters was initially challenged, as enterotypes were shown not to represent biologically discrete states (such as ABO blood types), but rather an analytical simplification derived from a multidimensional, gradual shift in genus profile [16]. Nevertheless, a consensus was eventually reached so that even though enterotypes may represent positions along a continuous gradient, the enterotype concept remained a useful tool for the stratification of gut bacterial communities [17]. Thus, “enterotyping” is a useful operational framework for stratifying individuals and exploring differential responses to dietary interventions based on distinct metabolic capacities, including differences in modulating nutrient metabolism [18] and energy harvest [19], which is crucial for body weight management. In this context, metabolite profiles may serve as complementary readouts to support and refine enterotype-based interpretations [16,17].

Whole milk is a primary dairy food and is commonly consumed as a part of the diet in many countries; yet, little is known about how it interacts with the gut microbiome and whether this can influence body weight homeostasis. To address this gap, we analyzed data from our previous RCT [9,10], treating the whole-milk lead-in phase as a short-term intervention and stratifying participants by their gut enterotype to assess enterotype-dependent variation in clinical parameters, exploring associations with metabolites in a hypothesis-generating manner following observation of enterotype-specific responses. Our findings reveal enterotype-dependent responses, highlighting their relevance for the design of personalized nutrition strategies.

## 2. Materials and Methods

### 2.1. Study Setting and Participants

This study was part of the FerMetS trial (ClinicalTrials.gov: NCT04755530), which enrolled 100 men with abdominal adiposity (body mass index, BMI, 28–45 kg/m^2,^ and waist circumference ≥ 102 cm), who habitually consumed dairy products. All participants underwent a one-month standardized lead-in phase, during which they were instructed to replace most of their habitual dairy food consumption (median of 372 g/day) with 400 g/day of whole milk (3.5% fat/100 g). Substitution was made on a group-level rather than an individual-level basis and was intended to be isocaloric, i.e., the lead-in phase aimed to maintain a stable body weight before starting the randomized interventions [9,10]. Whole milk was selected as it is the primary “prototypical” dairy food from which all other dairy products are subsequently produced.

### 2.2. Sample Collection and Clinical Variables

Fecal and urinary samples were collected at baseline (T0) and after the lead-in phase (T1). Body weight, BMI, waist circumference, hip circumference, and 24 h urinary nitrogen level were recorded at both time points. Fecal samples were collected at home, stored at −18 °C, and transported to the laboratory (in a cooler bag with cooler bricks) for storage at −80 °C within 24 h after collection. Urinary samples were collected in the morning of the day before the laboratory visit and stored in containers, which were kept at −18 °C before being transported to the laboratory (in a cooler bag with cooler bricks) for storage at −80 °C.

### 2.3. Metagenomics DNA Extraction, Library Construction, and Sequencing

DNA was extracted from 200 mg of fecal samples using the NucleoSpin Soil (250) kit (Macherey-Nagel, 52355 Düren, Germany, 740780.250M), following the manufacturer’s instructions. DNA quality was assessed with the Qubit 3.0 fluorometer using the Qubit 1X dsDNA Broad Range Assay Kit Q33266 (Thermo Fisher, Waltham, MA, USA). Library preparation for the next-generation sequencing (NGS) was performed using the MGIEasy FAST FS DNA Library Prep Set, 940-001196-00 (MGI Tech Co., Ltd., Shenzhen, China). In total, 300 ng of input DNA was subjected to the following steps: DNA fragmentation, end-repair and A-tailing, adapter ligation, PCR amplification, and final clean-up. Library quality was evaluated using both the Qubit 1X dsDNA HS Assay Kit Q33231 (Thermo Fisher, Waltham, MA, USA) and the Agilent High Sensitivity D1000 Assay Kits 5067–5584 and 5067–5585 (Agilent Technologies Santa Clara, CA, USA). After normalization, libraries were circularized with the MGIEasy Dual Barcode Circularization Kit 1000020570 (MGI Tech Co., Ltd., Shenzhen, China) and converted into DNA nanoballs (DNBs). Sequencing was carried out on the DNBSEQ-T7 platform (MGI Tech Co., Ltd., Shenzhen, China) using the DNBSEQ-T7RS High-throughput Sequencing Set FCL PE100, 940-000269-00 (MGI Tech Co., Ltd., Shenzhen, China), in accordance with the manufacturer’s protocol. All fecal samples were randomized before extraction and sequenced in the same run, which was distributed on two flow cells of the DNBSEQ-T7 platform. 

### 2.4. Metagenomic Data Processing, Taxonomic Profiling, KEGG Ortholog (KO) Annotation, and Metagenome-Assembled Genome (MAG) Reconstruction

The paired-end raw reads were first quality-trimmed using fastp (version 0.23.2) [20] with default parameters. The high-quality reads were then filtered to remove human-derived sequences (GRCh38_noalt_as) using Bowtie2 (version 2.2.3) [21], also with default parameters. Taxonomic annotation and profiling were performed using MetaPhlAn (version 4.1.0) [22] with default settings.

To evaluate functional changes during the study period, a gene set–based approach was employed. Filtered non-human reads were assembled using SPAdes (version 4.0.0, --meta) [23]. Open reading frames (ORFs) were predicted using Prodigal (version 2.6.3) [24], and redundant genes were removed using MMseqs2 (release_15-6f452) [25] at 95% sequence identity and 90% coverage. A total of 68,574,386 non-redundant genes were obtained. The relative abundance of each gene was calculated using CoverM (version 0.7.0) [26] with the parameters -m tpm, -p bwa-mem2, --min-read-aligned-length 75, --min-read-percent-identity 95, and --min-covered-fraction 0.2. Functional annotation was performed using eggNOG-mapper (version 2.1.12) [27], with a minimum identity threshold of 70% and a minimum coverage threshold of 50%. Gene abundances associated with the same KO identifier were summed to generate the KO profile.

The assembled scaffolds from each sample were processed using VAMB (version 4.1.3, -m 250) [28] to obtain metagenome-assembled genomes (MAGs). The quality of the MAGs was assessed using CheckM2 (version 1.0.2) [29] with default parameters. MAGs with >50% completeness and <10% contamination were retained and subsequently clustered using dRep (version 3.6.2, -sa 0.95, -nc 0.5) [30] to generate a non-redundant MAG catalog. A total of 1466 MAGs were obtained. Taxonomic assignment was performed using GTDB-Tk (version 2.3.2) [31] against the Genome Taxonomy Database (GTDB, release r214) [32]. The relative abundance of each MAG was calculated using CoverM (version 0.7.0) [26] with the same parameters used for gene abundance estimation.

Taxonomic annotation of selected genes was performed using NCBI web BLAST platform (https://blast.ncbi.nlm.nih.gov/Blast.cgi; accessed on 4 December 2025) for comparison to the core nucleotide database.

### 2.5. Gut Microbiome Community Typing (Enterotype Identification)

To address potential bias arising from limited sample size and differences in taxonomic profiling methods, we adopted a supervised microbiome community typing strategy. The MetaCardis Body Mass Index Spectrum (BMIS) cohort (*n* = 888; ENA project: PRJEB37249) [15] was used as a reference dataset. Enterotypes in the reference cohort were first identified. Genus-level profiles of the reference samples were generated using the same MetaPhlAn-based pipeline applied to the present project samples. Genus-level relative abundances were multiplied by 10^6^, rounded to integers, and clustered using the DirichletMultinomial package (v1.44.0) in R to identify enterotype [33].

To minimize biases introduced by different genus-profiling methods, we retained only the 430 reference samples that were consistently assigned to the same enterotype across three independent taxonomic profiling frameworks: molecular operational taxonomic unit (mOTU)-based [15], MAG-based [33], and our MetaPhlAn-based approach. These 430 reference samples formed four robust enterotypes (B1, B2, P and R) and were used as reference anchors. The present study samples were then combined with these reference samples and clustered to determine enterotype assignments. Among the 82 participants with available fecal samples collected both before (T0) and after (T1) the lead-in phase, enterotypes were assigned at each time point. Participants (*n* = 76) whose T0 and T1 samples were assigned to identical enterotypes were included for enterotype-specific features analysis (Supplementary Table S1). Six individuals exhibiting enterotype switching during the intervention were excluded to reduce analytical noise and preserve interpretability of enterotype-specific responses, as short-term whole milk consumption alone is unlikely to induce enterotype transitions; such changes may reflect unmeasured confounding influences. We acknowledge that restricting analyses to participants with stable enterotypes may introduce selection bias, potentially favoring individuals with less plastic or more resilient microbiome configurations. Moreover, the small number of participants exhibiting enterotype switching (*n* = 6) precluded meaningful comparative analyses of this subgroup. Consequently, the findings may not fully capture responses in individuals with more dynamic microbiome profiles.

### 2.6. Metabolome Analysis

Metabolomes of fecal and urinary samples were analyzed using nuclear magnetic resonance (NMR) spectroscopy. Samples were measured at 300 K on a 14 T Bruker Avance III spectrometer equipped with a TXI probe. One-dimensional ^1^H-NMR spectra were acquired using a NOESY presaturation pulse sequence, and selected samples were further examined with 2D JRES, HSQC, and COSY experiments. All spectra were Fourier-transformed, phase- and baseline-corrected, and referenced to TSP-d_4_ at δ 0.00. Metabolites were assigned using Chenomx and confirmed with 2D spectra when necessary; quantification was performed in Chenomx relative to the TSP-d_4_ internal standard. The detailed methodology and data have been published previously [10].

### 2.7. Assessment of Microbial Metabolism and Transport Capacity

Microbial enzymes involved in taurine biosynthesis, degradation, and transport were identified based on the KEGG pathway map00430 (taurine and hypotaurine metabolism) and the genes reported by Li et al. [34] and Wolf et al. [35]. Briefly, we assessed enzymes across three functional categories: (i) taurine biosynthesis (choloylglycine hydrolase; dimethylaniline monooxygenase (*N*-oxide forming)/hypotaurine monooxygenase; glutamate decarboxylase; sulfinoalanine decarboxylase/aspartate 1-decarboxylase; cysteate decarboxylation), (ii) taurine degradation pathways (bile acid-CoA:amino acid *N*-acyltransferase; taurine dioxygenase; taurine–2-oxoglutarate transaminase; taurine dehydrogenase), and (iii) taurine transport systems (TauABC). In addition, the bidirectional enzyme taurine–pyruvate aminotransferase was also included. The complete list of enzymes and their matched KOs, enzymatic reactions, and detection status is presented in Supplementary Table S2.

### 2.8. Statistical Analysis and Visualization

The normality of the data was assessed using the shapiro.test function in R (version 4.5.0). Both clinical variables and omics profiles deviated from a normal distribution.

Gut microbiome features (species, KOs, and MAGs) changes during the lead-in phase were assessed using MaAsLin2 [36] (normalization = “TSS”, transform = “LOG”, min_prevalence = 0.1), adjusting for age. Species profile is compositional, total sum scaling (TSS) normalization followed by log transformation (LOG) in MaAsLin2 analysis to mitigate variability across abundance levels. Differences in clinical variables and metabolite profiles were evaluated using paired Wilcoxon tests. Variables that showed significant within-enterotype changes were subsequently evaluated by comparing both their magnitudes of change and their before and after lead-in phase values between B1 and R enterotypes using unpaired Wilcoxon tests.

Differences in frequencies were assessed using the Chi-square test (counts > 5) or Fisher’s exact test (counts < 5).

All *p* values were adjusted for multiple comparisons using the Benjamini–Hochberg method, with an adjusted *p* value < 0.05 considered statistically significant. Unadjusted *p* values < 0.1 were interpreted as indicative of a statistical trend. Effect sizes were calculated to quantify the magnitude of change over time, with the Wilcoxon effect size computed by dividing the Z statistic by the square root of the number of observations (N) [37].

The relationship between body weight change and candidate variables was assessed using linear regression models using the lm () function in R. First, each variable was evaluated individually using simple linear regression models (Body weight change ~ variable) to identify variables associated with weight change (*p* < 0.05). Variables that met this criterion were then entered into a multiple linear regression model (Body weight change ~ variable_1_ + variable_2_ + … + variableₙ) to assess their independent contributions. Predictors that did not retain statistical significance in the multivariable context (*p* > 0.05) were removed to achieve a parsimonious final model. In addition, some variables were further evaluated as potential suppressors or modifying variables, even if not significant in univariable analyses. The multiple R^2^ and adjusted R^2^ values were used to assess the proportion of variance in body weight change explained by the regression model.

## 3. Results

### 3.1. Four Distinct Enterotypes at Baseline

**Figure 1:**
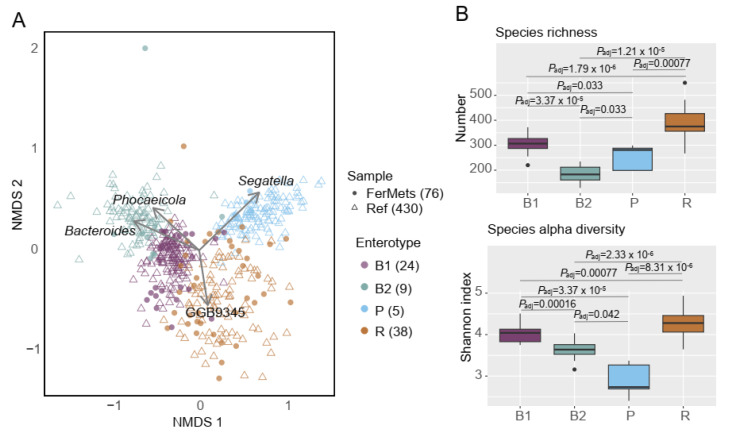
Four enterotypes at baseline. (**A**) NMDS plot of genus-level profiles from 76 baseline FerMets samples and 430 reference samples from the BMIS cohort. The taxonomic classification of genus GGB9345 is: *k__Bacteria*; *p__Firmicutes*; *c__CFGB10477*; *o__OFGB10477*; *f__FGB10477*; *g__GGB9345*. (**B**) Species richness and alpha diversity across enterotypes at baseline. Differences were assessed using the Wilcoxon test. *p* values were adjusted for multiple comparisons using the Benjamini–Hochberg method.

Out of 100 men with abdominal adiposity who were randomized to the original RCT, fecal samples were collected from 92, of whom 82 had metagenomic data available both before (T0) and after (T1) the lead-in phase. Among these, 76 individuals exhibited a stable enterotype during the one-month lead-in whole milk phase: B1 (*n* = 24), B2 (*n* = 9), P (*n* = 5), and R (*n* = 38) (Figure 1A and Supplementary Table S1). Based on these 76 participants with stable enterotypes, we found the B1 and B2 enterotypes were primarily driven by the genera *Phocaeicola* and *Bacteroides*, while the P and R enterotypes were dominated by *Segatella* (a genus recently separated from *Prevotella*) [38], and GGB9345 (Firmicutes genus annotated in the MetaPhlAn database), respectively. A progressive increase in species richness was observed across fecal samples from participants with B2, P, B1, and R enterotypes, whereas an increase in Shannon index was observed in the order P, B2, B1, and R (Figure 1B).

### 3.2. Baseline Features of B1 and R Participants

Due to the limited number of participants in the B2 (*n* = 9) and P (*n* = 5) enterotypes, subsequent analyses were restricted to individuals classified as B1 and R. No significant differences in demographic variables or habitual dairy consumption were observed between participants with these two enterotypes (Supplementary Table S3).

Fecal dimethylamine levels were significantly higher in R compared to B1 individuals (*P*_adj_ = 0.037) (Supplementary Figure S1 and Supplementary Table S4). In addition, the levels of several metabolites tended to differ between B1 and R individuals, including urinary taurine (*p* = 0.0024, *P*_adj_ = 0.088), fecal alanine (*p* = 0.0094, *P*_adj_ = 0.11), and fecal tryptophan (*p* = 0.0067, *P*_adj_ = 0.11) (Supplementary Figure S1 and Supplementary Tables S4 and S5).

Next, the metabolic potentials of the microbiomes at baseline were examined. We found that genes involved in microbiome dimethylamine metabolism were enriched in R participants (Supplementary Table S6), which may partially explain the higher fecal dimethylamine levels in R individuals.

### 3.3. Features Altered During the Lead-In Phase

**Figure 2:**
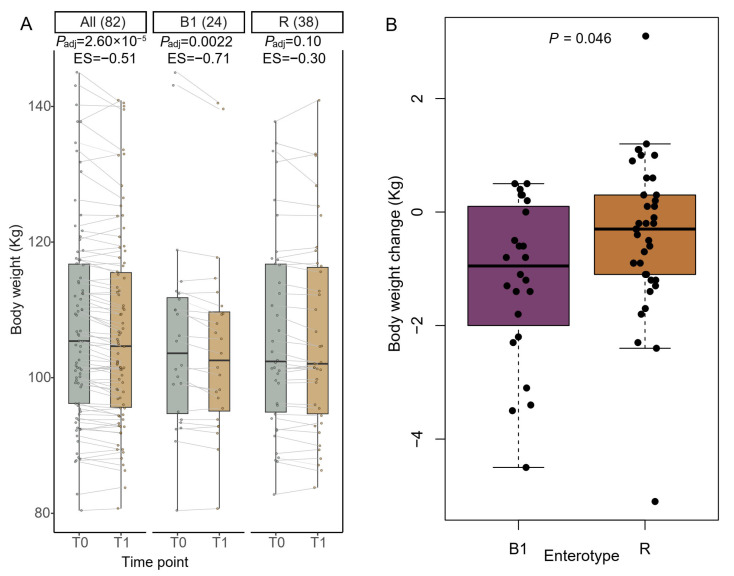
Changes in body weight during a one-month whole-milk lead-in phase. (**A**) Body weight before and after the lead-in phase in B1 and R individuals. (**B**) Body weight loss in B1 and R individuals. Within-enterotype differences were assessed using paired Wilcoxon tests, and *p* values were adjusted for multiple comparisons using the Benjamini–Hochberg method. Between-enterotype differences in body weight loss were evaluated using the unpaired Wilcoxon test.

Despite the anticipated weight stability during the whole-milk lead-in phase, we observed a reduction in body weight in the entire cohort (Figure 2A). Upon stratification according to enterotypes, we observed that individuals with the B1 enterotype exhibited a significantly reduced body weight (*P*_adj_ = 0.0022), with an average loss of 1.18 kg, higher than the observed 0.40 kg reduction in R individuals (*p* = 0.046) (Figure 2A,B). No other differences in phenotypic measures were observed between enterotypes after the lead-in phase (Supplementary Table S7). Accordingly, a significant decrease in BMI was observed exclusively in B1 individuals (*P*_adj_ = 0.0034), with an average reduction of 0.38 kg/m^2^, which was larger than (*p* = 0.035) the 0.12 kg/m^2^ reduction observed in the R group (Supplementary Table S8 and Supplementary Figure S2A). A paradoxical significant reduction in waist circumference was observed in R individuals (*P*_adj_ = 0.037); however, the magnitude of change did not differ significantly between B1 and R individuals (*p* = 0.70) (Supplementary Table S8 and Supplementary Figure S2B).

We next assessed changes in gut microbiome features. Whereas alpha diversity and richness based on species profiles did not change significantly in either B1 or R individuals, indicating overall stability of the gut microbiome community structure during the lead-in phase. At the function level, both KO richness and KO alpha diversity remained stable in B1 individuals (*P*_adj_ > 0.1). Although KO richness tended to increase in R participants (*p* = 0.014, *P*_adj_ = 0.085) (Supplementary Figure S3), none of KOs reached statistical significance after adjustment for multiple comparisons (Supplementary Table S9).

**Figure 3:**
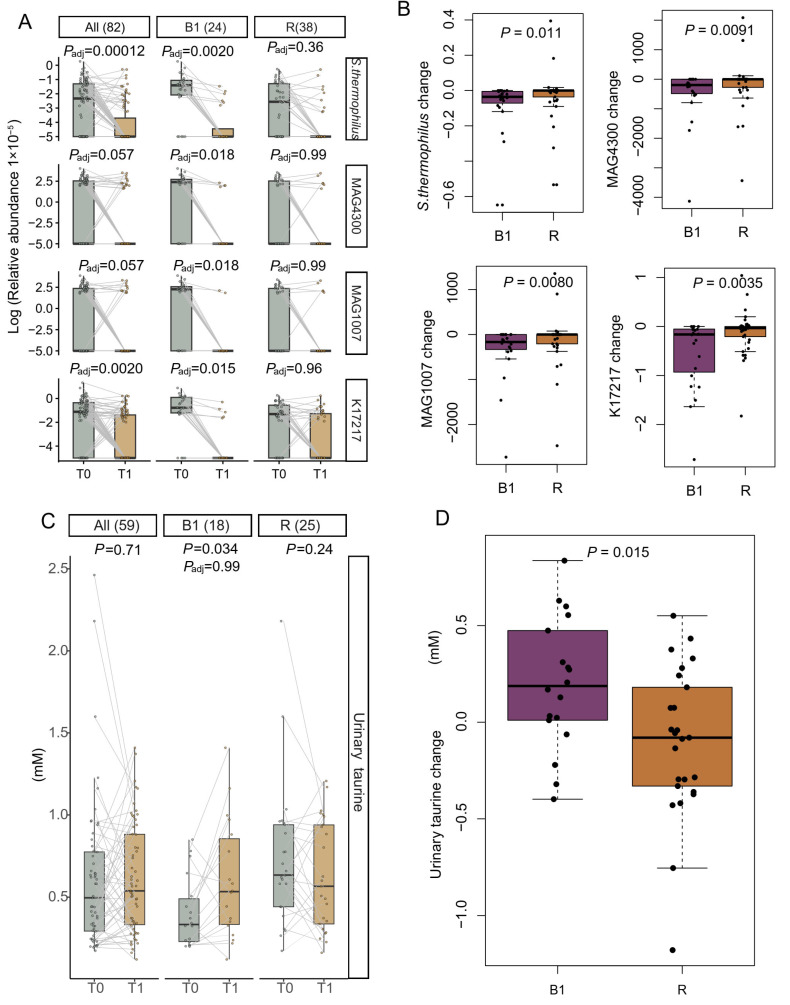
Changes in the relative abundance of *Streptococcus thermophilus*, KEGG ortholog (KO) K17217, and urinary taurine levels during the lead-in phase. (**A**) The relative abundance of *S. thermophilus*, two *S. thermophilus* metagenome-assembled genomes (MAGs), and K17217 before and after the lead-in phase. (**B**) The relative abundance changes in *S. thermophilus*, two *S. thermophilus* MAGs, and K17217 in B1 and R individuals. (**C**) Urinary taurine levels before and after the lead-in phase. (**D**) Urinary taurine changes in B1 and R individuals. K17217: Cystathionine gamma-lyase/homocysteine desulfhydrase. Within-enterotype differences in species, KO, and MAG profiles were evaluated using MaAsLin2. Within-enterotype differences in urinary taurine levels were assessed using paired Wilcoxon tests. *p* values were adjusted for multiple comparisons using the Benjamini–Hochberg method. Between-enterotype differences in feature changes were evaluated using unpaired Wilcoxon tests.

A significant reduction in the relative abundance of *Streptococcus thermophilus* (*P*_adj_ = 0.0020), two *S. thermophilus* MAGs (MAG4300: *P*_adj_ = 0.018 and MAG1007: *P*_adj_ = 0.018), and the gene encoding cystathionine gamma-lyase/homocysteine desulfhydrase (K17217) (*P*_adj_ = 0.015) was observed exclusively in B1 participants (Figure 3A). Accordingly, the magnitude of changes for these features also differed significantly between B1 and R individuals (Figure 3B). It is of note that both B1 (79.2% to 25.00%) and R individuals (57.89% to 23.68%) exhibited a marked reduction in the prevalence of *S. thermophilus* (Supplementary Table S10) during the lead-in phase.

To investigate the source of the observed decrease in the relative abundance of the gene encoding cystathionine gamma-lyase/homocysteine desulfhydrase (K17217), we interrogated 105 genes encoding this enzyme. These genes were primarily assigned to the species *S. thermophilus* (26.66%), *Lacticaseibacillus paracasei* (14.28%), *Lactococcus lactis* (8.57%), and *Lactobacillus acidophilus* (8.57%) (Supplementary Table S11). Notably, only the abundance of genes derived from *S. thermophilus* decreased during the lead-in phase, indicating that the reduction in K17217 was partly driven by a decrease in *S. thermophilus*.

We also assessed the relative abundance of *Bifidobacterium*, previously reported to increase in abundance following whole milk consumption [39,40,41], but neither B1 participants (*p* = 0.92) nor R participants (*p* = 0.97) exhibited statistically significant changes (Supplementary Table S10).

The fecal level of three metabolites—alanine, tryptophan, and dimethylamine—tended to differ between B1 and R individuals at baseline, and these differences were attenuated after the lead-in period (Supplementary Figure S1). No significant changes in fecal metabolites were observed during the lead-in phase in either B1 individuals or R individuals (Supplementary Table S12). Among the urinary metabolites, although the within-enterotype change in taurine levels was not statistically significant after correction for multiple comparisons (B1: *p* = 0.034, *P*_adj_ = 0.99; R: *p* = 0.24) (Figure 3C and Supplementary Table S13), the magnitude of changes were significantly greater in B1 than in R individuals (*p* = 0.015) (Figure 3D), and baseline difference trend between B1 and R individuals (*p* = 0.0024; *P*_ad_ = 0.088) vanished after the lead-in phase (*p* = 0.61) (Supplementary Figure S1), suggesting a potential enterotype-specific response in taurine dynamic during the lead-in phase.

### 3.4. Enterotypes and Body Weight Change

We applied multiple linear regression to assess the independence of enterotype as a predictor of body weight change and to identify additional contributing factors. A total of 29 variables were considered, including enterotype, baseline enterotype-associated features (baseline species richness, species Shannon diversity, and metabolites that differed between enterotypes), and lead-in phase enterotype-associated features (changes in K17217, MAG4300, MAG1007, *S. thermophilus,* and urinary taurine level). In addition, baseline total energy intake and dietary variables, including dairy product intake and macronutrient consumption, were also included, as these factors may also be associated with body weight change.

We first employed simple linear regression models to screen each variable individually. B1 enterotype (*p* = 0.033) and baseline body weight (*p* = 0.019) were inversely associated with body weight change, where baseline fermented dairy consumption showed a trend toward association (*p* = 0.084) (Supplementary Table S14). When these three variables were included in a multivariable linear regression model, the associations with enterotype (*p* = 0.021) and baseline body weight (*p* = 0.032) remained robust, whereas the association with baseline fermented dairy consumption was no longer observed (*p* = 0.16) (M0, Supplementary Table S14). Therefore, baseline fermented dairy consumption was excluded from the final prediction model, while enterotype and baseline body weight were retained (M1, Supplementary Table S14).

Although urinary taurine level change was not associated with body weight change in univariate analyses (*p* = 0.44) (Supplementary Table S14), given its potential role as a suppressor or effect-modifying variable, urinary taurine level change was subsequently included in the multivariable model alongside enterotype and baseline body weight. In this adjusted model, urinary taurine level change became positively associated with body weight change (*p* = 0.021) (Table 1), highlighting that the relationship between body weight change and urinary taurine level change may be enterotype-dependent.

**Figure 4:**
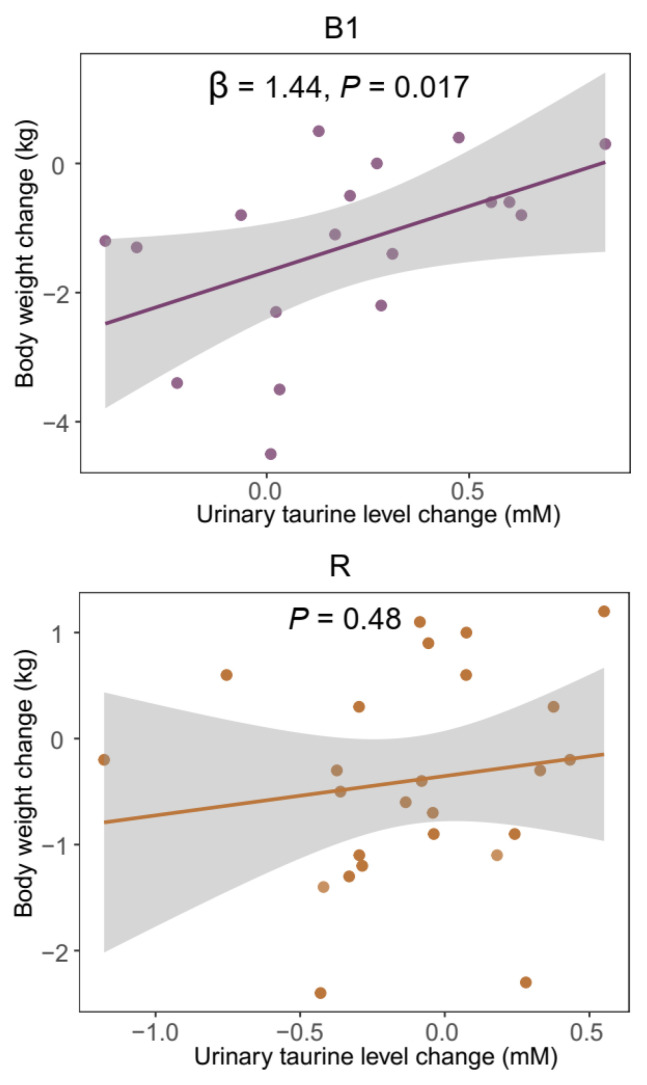
Association between body weight changes and urinary taurine level changes in B1 and R individuals. The gray shaded area indicates the 95% confidence interval. In B1 individuals, body weight change was positively associated with urinary taurine level change (*p* = 0.017) after adjusting for baseline body weight (body weight change ~ urinary taurine level change + baseline body weight). In R individuals, no significant association was observed (*p* = 0.48). Linear regression models were used to identify predictors of weight loss. Full regression results for B1 and R individuals are provided in Supplementary Tables S15 and S16, respectively.

We further explored the relationship between body weight change and urinary taurine level change within each enterotype. Among B1 individuals, body weight change was inversely associated with baseline body weight (*p* = 0.000039) and positively associated with urinary taurine change (*p* = 0.017) (Figure 4 and Supplementary Table S15). In contrast, within R individuals, body weight change showed no significant association with either baseline body weight (*p* = 0.59) or urinary taurine level change (*p* = 0.48) but was inversely associated with baseline species Shannon diversity (*p* = 0.014) (Figure 4 and Supplementary Table S15).

### 3.5. Gut Microbiome Potential for Taurine Metabolism and Transport

To explore the potential contribution of the gut microbiome to the change in urinary taurine level during the lead-in phase, we assessed the potential for taurine metabolism and transport capacity in the B1 and R microbial communities. Genes encoding two taurine biosynthesis enzymes (choloylglycine hydrolase and glutamate decarboxylase), two taurine degradation enzymes (taurine dioxygenase and taurine–2-oxoglutarate transaminase), one bidirectional taurine-metabolizing enzyme (taurine–pyruvate aminotransferase), and three components of the taurine transport system (substrate-binding, permease, and ATP-binding proteins) were examined (Supplementary Figure S5). Among these genes, no significant alterations in relative abundance were observed within either B1 or R microbial communities during the lead-in phase (Supplementary Table S17). We also examined the taurine metabolism potential of *S. thermophilus*. For the two *S. thermophilus* MAGs (MAG4300 and MAG1007), none of the five taurine metabolism enzymes or three taurine transporter components were identified (Supplementary Table S18), suggesting that the gut microbiome did not contribute to inter-enterotype variation in urinary taurine level change through direct taurine metabolism and transporter.

### 3.6. The Correlation Between S. thermophilus Change and Urinary Taurine Level Change

We next examined whether changes in the abundance of *S. thermophilus* were associated with changes in urinary taurine level. Linear regression models were performed to identify predictors of urinary taurine level change. Body weight change showed no significant association with urinary taurine level change (*p* = 0.44). Unexpectedly, the effect of enterotype was no longer evident in the multivariable models (*p* = 0.77), whereas baseline urinary taurine level and *S. thermophilus-related* features change remained robustly associated with urinary taurine level change (Supplementary Table S19).

We then shifted the outcome variable to change in MAG4300 relative abundance to assess whether the association between urinary taurine level change and *S. thermophilus* change was robust. Unexpectedly, in multivariate models, changes in MAG4300 relative abundance were strongly associated only with baseline MAG4300 relative abundance (*p* = 1.65 × 10^−20^) and showed no association with either enterotype (*p* = 0.82) or urinary taurine level change (*p* = 0.28) (Supplementary Table S20).

## 4. Discussion

Dairy consumption has been proposed to aid in the management of overweight and obesity. However, the outcomes have been controversial, particularly across populations with differing habitual dairy intake [42,43,44,45,46]. The present study offers a potential explanation for this variability, suggesting that the gut microbiome may act as a potentially contributing factor. We found that men with abdominal adiposity carrying the B1 enterotype experienced body weight loss during a one-month whole-milk lead-in phase, accompanied by a decrease in the relative abundance of *S. thermophilus*. Urinary taurine levels also changed in an enterotype-specific manner. These observations were unexpected, as the lead-in phase was designed to maintain body weight rather than induce weight change, raising concerns for the design of appropriate lead-in strategies in dietary intervention studies.

We then explored potential associated factors underlying the observed reduction in body weight. Enterotype emerged as a potential independent predictor of weight change, and among individuals with the B1 enterotype, the change in urinary taurine level was positively associated with body weight change, highlighting taurine as a potentially contributing factor associated with weight loss. Taurine is a sulfur-containing amino acid that is present only in low amounts in whole milk [47] but abundant in seafood, particularly scallops [48]. Unlike other amino acids that serve as energy sources, humans lack taurine-degrading enzymes, so taurine is retained within tissues, primarily serving as an intracellular osmolyte, rather than being metabolized [49]. Studies have shown that taurine supplementation can lead to significant weight reduction or improvements in lipid metabolism [50,51,52,53,54]. The underlying mechanisms may involve inhibition of hepatic lipid biosynthesis by suppressing the expression of fatty acid synthase [55], upregulation of expression of genes involved in thermogenesis in brown adipose tissue [56], browning of white adipose tissue [57], improving the function of pancreatic islet β-cells through alleviation of oxidative stress and apoptosis [58], and stimulation of secretion of glucagon-like peptide-1 (GLP-1) from neuroendocrine L cells [59]. Notably, in the present study, we observed a positive association between urinary taurine level changes and body weight changes in B1 individuals, where individuals exhibiting smaller increases in urinary taurine levels tended to experience greater body weight loss. Although seemingly counterintuitive, this pattern suggests that tissue taurine retention, rather than urinary excretion, may be associated with weight loss. Smaller increases in urinary taurine levels may reflect greater taurine retention within host tissues, consistent with a previous report linking elevated taurine concentrations in adipose tissue to enhanced fat loss [60]. Alternatively, body weight loss and taurine levels may be linked in a bidirectional manner, with changes in body weight potentially influencing taurine dynamics [60]. However, due to the lack of comprehensive taurine-level data in the current study, this relationship could not be tested within the present dataset and therefore warrants further investigation.

We also explored the potential associations between gut microbiome and urinary taurine levels. In the present study, both at baseline and during the lead-in phase, urinary taurine levels showed an enterotype-specific variation, highlighting a potential role of enterotype or the gut microbiome in regulating taurine homeostasis. However, the overall microbiome-associated taurine metabolic capacity did not change during the lead-in phase in either B1 or R individuals. In contrast, we observed an association between *S. thermophilus* and urinary taurine levels, characterized by a negative correlation between change in urinary taurine levels and changes in *S. thermophilus* relative abundance. This observation is consistent with a previous study reporting that administration of specific *S. thermophilus* strains to mice for 28 days markedly reduced serum taurine concentrations, with strain DQHXNQ38M61 decreasing serum taurine levels by 44% compared with controls [61]. As the taurine metabolizing enzymes were not identified in two recovered *S. thermophilus* MAGs, these findings raise the possibility that *S. thermophilus* may influence host taurine homeostasis indirectly, for instance, through modulation of host sulfur metabolism [61]. Notably, microbiome-associated taurine metabolic capacity was evaluated at the gene level, which provides lower confidence in functional activity than expression-based measurements. Moreover, although changes in urinary taurine levels did not predict changes in *S. thermophilus* abundance in the present study, the relationship between *S. thermophilus* and taurine may not be unidirectional. Taurine itself could potentially influence gut microbiome composition, including *S. thermophilus* abundance, through mechanisms such as bile acid conjugation and deconjugation [60,62]. In addition, it should be noted that urinary taurine dynamics are also influenced by non-microbial factors, including dietary intake, endogenous biosynthesis, and renal transport capacity, which may also contribute to the observed variability.

The reduction in *S. thermophilus* relative abundance was predicted only by the baseline *S. thermophilus* level. Therefore, aside from any potential association with taurine dynamics, another plausible explanation for the decline in *S. thermophilus* abundance may be that participants replaced their habitual consumption of yogurt or cheese, which typically contain *S. thermophilus*, with whole milk during the lead-in phase (as instructed), thereby reducing dietary intake of this bacterial species. In addition, given evidence that dietary whey protein can influence gut phage dynamics [63], phage activity may also have contributed to the observed reduction. This reduction may exhibit a “taurine supplementation” like effect, potentially increasing taurine availability, with one component retained in host tissues and associated with body weight loss, and another component reflected as increased urinary taurine excretion. The lower urinary taurine level and higher prevalence of *S. thermophilus* observed in baseline B1 individuals are consistent with this interpretation. Although *S. thermophilus* prevalence also declined in R individuals, this reduction was not associated with a significant change in body weight or urinary taurine level. This discrepancy may reflect the greater microbial diversity of the R microbiome community, which could buffer metabolic effects, or an insufficient magnitude of change to elicit measurable host phenotypic or metabolic responses.

There are several limitations to the present study. Notably, interactions among body weight regulation, taurine homeostasis, and gut microbiome balance are complex and potentially multidirectional [60]. Important confounding factors related to body weight regulation, such as total energy intake, physical activity, and overall dietary composition beyond the prescribed dairy intervention, were not measured or incorporated into the predictive models. Although random variation in these unmeasured factors is unlikely to align systematically with enterotype classification, the present findings should be regarded as exploratory, and further validation in well-controlled, prospective studies is warranted.

## 5. Conclusions

In summary, our findings suggest that individuals with the B1 enterotype may be more prone to weight loss in response to whole milk consumption, and that this may be associated with *S. thermophilus* reduction and taurine redistribution. Importantly, this work underscores the potential value of incorporating enterotype stratification into personalized nutrition strategies [64,65] and highlights the need for careful consideration of the lead-in phase design in dietary intervention studies.

## Figures and Tables

**Table 1:** Body weight change-related variables.

Predictor	Estimate	Std. Error	T Value	*p* Value	R^2^	Adjusted R^2^
(Intercept)	2.51	1.24	2.02	0.050	0.37	0.33
Baseline body weight	−0.038	0.011	−3.27	0.0023		
Enterotype B1	−1.07	0.34	−3.12	0.0034		
Urinary taurine level change	1.04	0.43	2.40	0.021		

## Data Availability

Data access and handling complied with the European General Data Protection Regulation (GDPR). Data will be shared following review and approval by the ethical and scientific boards. The shotgun next-generation sequencing reads (with human DNA removed) have been deposited in the Sequence Read Archive (SRA) under BioProject accession number PRJNA1308307.

## Supplementary Materials

The following supporting information can be downloaded at: https://www.mdpi.com/article/10.3390/nu18040563/s1, Supplementary Figure S1. Levels of four metabolites that differed in abundance between B1 and R individuals at baseline and their levels after the lead-in phase. Supplementary Figure S2. BMI changes and waist circumference changes during the lead-in phase of B1 and R individuals. Differences between enterotypes were assessed using unpaired Wilcoxon tests. Supplementary Figure S3. Changes in species level and KEGG Ortholog (KO) richness and alpha diversity during the one-month lead-in phase. Within-enterotype differences were analyzed by paired Wilcoxon test. P values were adjusted for multiple comparisons using the Benjamini–Hochberg method. Supplementary Figure S4. Changes in urinary lactose level during the lead-in phase in B1 and R individuals. Differences between enterotypes were assessed using unpaired Wilcoxon tests. Supplementary Figure S5. Schematic overview of taurine metabolism and transport. Supplementary Table S1. Enterotype classification of participants at baseline (T0) and after the lead-in phase (T1). Supplementary Table S2. KEGG Orthologs (KOs) involved in taurine metabolism and transport. Supplementary Table S3. Baseline demographic characteristics and habitual dairy intake of B1 and R participants. Supplementary Table S4. Comparison of baseline fecal metabolites between B1 and R participants. Supplementary Table S5. Comparison of baseline urinary metabolites between B1 and R participants. Supplementary Table S6. Comparison of baseline KEGG Orthologs (KOs) between B1 and R participants. Supplementary Table S7. Demographic characteristics of participants after the lead-in phase. Supplementary Table S8. Changes in demographic characteristics during the lead-in phase. Supplementary Table S9. Changes in KEGG Orthologs (KOs) prevalence in R participants during the lead-in phase. Supplementary Table S10. Changes in gut microbial species, metagenome-assembled genomes (MAGs), and KEGG Orthologs (KOs) during the lead-in phase. Supplementary Table S11. Taxonomic annotation of KEGG Ortholog K17217 encoding genes. Supplementary Table S12. Changes in fecal metabolites during the lead-in phase. Supplementary Table S13. Changes in urinary metabolites during the lead-in phase. Supplementary Table S14. Associations between candidate variables and body weight changes in all participants. Supplementary Table S15. Associations between candidate variables and body weight changes in B1 participants. Supplementary Table S16. Associations between candidate variables and body weight changes in R participants. Supplementary Table S17. Comparison of the relative abundance of taurine metabolism and transport related KEGG Orthologs (KOs) during the lead-in phase. Supplementary Table S18. Taurine-related KEGG Orthologs (KOs) detected in two *Streptococcus thermophilus* metagenome-assembled genomes (MAGs). Supplementary Table S19. Associations between candidate variables and changes in urinary taurine levels in all participants. Supplementary Table S20. Associations between candidate variables and changes in *Streptococcus thermophilus* metagenome-assembled genome MAG4300 abundance in all participants.

## Abbreviations

The following abbreviations are used in this manuscript:

MAG	Metagenome-assembled genomes
KEGG	Kyoto Encyclopedia of Genes and Genomes
KO	KEGG Ortholog
