# Supplementary material for: Associations Between Gut Microbiome Enterotypes and Body Weight Change During Whole Milk Consumption
Source: Nutrients. 2026 Feb 9;18(4):563. doi: 10.3390/nu18040563 (PMC12943088; doi:10.3390/nu18040563)
Supplement: Supplementary file 1 [file nutrients-18-00563-s001.zip › Supplementary Figure Legends.pdf]

Supplementary Figure S1. Levels of four metabolites that differed in abundance between B1 and R individuals at baseline and their levels after the lead-in phase.

Supplementary Figure S2. BMI changes and waist circumference changes during the lead-in phase of B1 and R individuals. Differences between enterotypes were assessed using unpaired Wilcoxon tests.

Supplementary Figure S3. Changes in species level and KEGG Ortholog (KO) richness and alpha diversity during the one-month lead-in phase. Within-enterotype differences were analyzed by paired Wilcoxon test. P values were adjusted for multiple comparisons using the Benjamini–Hochberg method.

Supplementary Figure S4. Changes in urinary lactose level during the lead-in phase in B1 and R individuals. Differences between enterotypes were assessed using unpaired Wilcoxon tests.

Supplementary Figure S5. Schematic overview of taurine metabolism and transport.
